# Methodological Concerns in AI Medical Education Frameworks

**DOI:** 10.2196/101696

**Published:** 2026-07-06

**Authors:** Syeda Urooj Fatima, Emaan Fatima, Ragni Kumari

**Affiliations:** 1 Dow University of Health Sciences, E-120 Al-Falah Housing Project, Malir Halt, Karachi, Karachi, Sindh, 75210, Pakistan

**Keywords:** artificial intelligence, medical education, machine learning, adaptive learning systems, future implications

We read with great interest the recently published viewpoint by Izquierdo-Condoy et al [1] on artificial intelligence (AI) in medical education and their proposed FACETS (form, application, context, instructional mode, technology, SAMR [substitution, augmentation, modification, redefinition]) framework. While we commend the authors for a timely overview, we wish to highlight two concerns, the resolution of which may further strengthen the article.

First, the authors cite Abdelwanis et al [2] to support the claims about AI-driven educational inequities in low- and middle-income countries (LMICs). However, the cited study presents a conceptual bowtie analysis of automation bias conducted at an institution in a high-income country (the United Arab Emirates) with no LMIC populations, data, or contextual analysis—and explicitly excludes human or animal participants, further restricting its empirical validity. Therefore, citing this reference to support an LMIC-specific argument is a form of citation distortion; that is, it is deploying a source beyond the boundaries of its actual scope. This is not a minor citation error. Equity arguments built on mismatched evidence invite dismissal by reviewers and policymakers —weakening advocacy where it is most needed. If LMIC-specific AI education gaps cannot be substantiated with direct evidence, then there is a real risk that resource allocation decisions and curriculum reform efforts in low-income settings will be deprioritized or delayed. This matters because LMIC disparities in AI medical education are real and widening: Ong et al [3] demonstrated that LMIC experts were significantly less likely to consider AI learning outcomes mandatory compared to high-income country peers—a finding that would have directly and legitimately supported the authors’ argument.

Second, the authors retrospectively mapped the FACETS framework onto 2 single-institution feasibility studies by authors from well-resourced European universities—Holderried et al [4] (n=106 conversations in Tübingen) and Luordo et al [5] (n=96 students in Madrid), while acknowledging that these studies did not explicitly use FACETS. Critically, both studies revealed unresolved AI performance gaps: 8 of 45 feedback categories showed low concordance with human raters in Holderried et al [4], and AI scoring was systematically stricter than expert grading, by 3.51 points, in Luordo et al [5]—concerns that FACETS fails to address. Retrospective application shows descriptive compatibility, not predictive validity —a key distinction for responsible advocacy. A framework that only describes the past cannot help institutions anticipate AI failures. In high-stakes contexts like objective structured clinical examination (OSCE) grading or history-taking, such failures directly harm assessment integrity and patient safety preparation. If institutions in diverse, resource-limited settings adopt FACETS based on this evidence, they risk implementing AI-driven assessments calibrated to well-resourced European contexts, potentially disadvantaging students in settings where infrastructure, language, and clinical exposure differ substantially. Before FACETS is supported as a guiding standard, it must undergo multi-institutional, cross-cultural validation.

As AI integrates into medical education, frameworks must be evaluated across diverse settings. Without rigorous validation, weak evidence will undermine equity efforts, and premature framework adoption will shape curricula that are difficult to reverse — ultimately compromising the preparedness of future physicians in the very places that need improvement most.

## Abbreviations

AI: artificial intelligence
FACETS: form, application, context, instructional mode, technology, [substitution, augmentation, modification, redefinition]
LMIC: low- and middle-income country
OSCE: objective structured clinical examination
